# Intrahost variations in the envelope receptor-binding domain (RBD) of HTLV-1 and STLV-1 primary isolates

**DOI:** 10.1186/1742-4690-3-29

**Published:** 2006-05-25

**Authors:** Felix J Kim, Madakasira Lavanya, Antoine Gessain, Sandra Gallego, Jean-Luc Battini, Marc Sitbon, Valérie Courgnaud

**Affiliations:** 1 Institut de Génétique Moléculaire de Montpellier (IGMM), 1919 Rte de Mende, F-34293 Montpellier Cedex 5, France; CNRS, UMR5535, Montpellier, France; Université Montpellier 2, IFR122, Montpellier, France; 2 Institut Pasteur, Département de Virologie, 28 rue du Dr Roux, 75015 Paris, France; Unité d'Epidémiologie et Physiopathologie des Virus Oncogènes, Paris, France; CNRS, URA 1930, Paris, France; 3 Laboratory of Human Lymphotropic Viruses, Cordoba, Argentina; Virology Institute, School of Medicine, Cordoba, Argentina; National University of Cordoba, Cordoba, Argentina; 4 Memorial Sloan-Kettering Cancer Center 1275 York Ave, New York, NY, 10021, USA

## Abstract

Four primate (PTLV), human (HTLV) and simian (STLV) T-cell leukemia virus types, have been characterized thus far, with evidence of a simian zoonotic origin for HTLV-1, HTLV-2 and HTLV-3 in Africa. The PTLV envelope glycoprotein surface component (SUgp46) comprises a receptor-binding domain (RBD) that alternates hypervariable and highly conserved sequences. To further delineate highly conserved motifs in PTLV RBDs, we investigated the intrahost variability of HTLV-1 and STLV-1 by generating and sequencing libraries of DNA fragments amplified within the RBD of the SUgp46 *env *gene. Using new and highly cross-reactive *env *primer pairs, we observed the presence of Env quasispecies in HTLV-1 infected individuals and STLV-1 naturally infected macaques, irrespective of the clinical status. These intrahost variants helped us to define highly conserved residues and motifs in the RBD. The new highly sensitive *env *PCR described here appears suitable for the screening of all known variants of the different PTLV types and should, therefore, be useful for the analysis of seroindeterminate samples.

## Findings

Human T-cell lymphotropic viruses (HTLV) and their simian T-cell lymphotropic virus (STLV) counterparts belong to the *Retroviridae *family and are globally referred to as primate T-cell lymphotropic viruses (PTLV). Thus far, four distinct groups of PTLV have been discovered: PTLV-1, -2 and -3 include both human (HTLV-1, -2, -3) and simian (STLV-1, -2, -3) viruses while the fourth type (HTLV-4) has only been described in humans [1-3]. HTLV-1 is a persistent virus, infecting 15–25 million people worldwide, the majority of whom remain asymptomatic their entire life. However, HTLV-1 is the etiological agent of a malignant CD4 lymphoproliferation (adult T-cell leukemia [ATL]) [4] and a chronic progressive neuromyelopathy (tropical spastic paraparesis/HTLV-1-associated myelopathy [TSP/HAM]) [5,6]. In addition, HTLV-1 has been shown to be associated with a range of other inflammatory diseases [7,8]. Transmission of PTLV occurs predominantly from mother to child by breast feeding [9] and by sexual or blood contacts [10,11].

The close relationship between HTLV and STLV suggests a simian origin for HTLV. The HTLV-I strains can be classified into six different subtypes according to their geographic origin [2]. Moreover, phylogenetic analyses of the global spread of PTLV-1 strains has shown that some HTLV-1 strains are closely related to STLV-1, suggesting the occurrence of multiple cross-species transmissions between primates and humans and also between different primate species [12].

Unlike other retroviruses, which in general show a high rate of nucleotide substitutions, PTLV exhibit a remarkable genetic stability [13]. This is generally attributed to the fact that these viruses replicate *in vivo *mainly via clonal expansion of infected cells [14-17]. Despite this low level of variability of HTLV-1 (from 1% to 8% between strains [18-20], a few PCR-based variability studies have shown some intrastrain variability in several parts of the viral genome, such as the LTR U3, *tax *or *env *[18,21-23]. On the other hand, almost no genetic variation has been observed in the 5'end of HTLV-1 *env *in samples obtained from 2 asymptomatic patients [24]. Thus, the extent of intrahost genetic diversity in HTLV-infected individuals is not well known.

The extracellular surface component (SU) of retroviral envelopes is involved in cellular tropism, target cell infection, and induction of host viral immunity. For any retrovirus, the SU exhibits the highest level of protein variability [25]. The prototypic Gammaretrovirus MLV SU comprises several variable regions that confer receptor binding properties which are distinctive of MLV subgroups [26]. The HTLV-1 envelope glycoprotein consists of an SUgp46 associated to a TMgp21 transmembrane component. HTLV SU has been shown to have a modular structure similar to that of MLV SU [27,28] and like all identified gammaretrovirus envelopes [29,30] it recognizes a multimembrane-spanning nutrient-transporter as a receptor. The first 160 amino acids of the 291 residue-long mature HTLV-1 SU have been shown to contain the HTLV Env receptor binding domain (RBD) and to direct binding to the glucose transporter GLUT1 shown to be a HTLV-1 and 2 receptor [28,31,32]. This binding involves the carboxy terminal 6th extracellular loop (ECL6) in GLUT1, whereas other receptor determinants in ECL1 and ECL5 of GLUT1 appear to modulate post-binding viral entry events [32].

In order to delineate conserved motifs that are likely to be involved in binding or post-binding events, we have investigated the intrahost variability of HTLV-1 and STLV-1 RBD by sequencing intrahost libraries of DNA fragments amplified from the RBD-encoding part of the *env *gene.

DNA directly isolated from blood samples obtained from three unrelated infected individuals, one asymptomatic seropositive donor, one ATL patient, and one TSP/HAM patient, were used to derive a fragment library of SU RBD and *tax *amplicons. In parallel, we amplified the equivalent regions in samples obtained from 2 STLV-1 naturally-infected Celebes macaques (*Macaca tonkeana*) from Indonesia [33].

Using a multiple envelope alignment of all available PTLV strain types, we designed degenerate PCR primers spanning the RBD in order to allow the detection of all PTLV *env*-like sequences. We delineated a 195 nt sequence surrounding the *env *gene codon corresponding to Tyr114 in the HTLV-1 SU RBD, previously shown to be a critical determinant for HTLV Env receptor binding [31]. This PTLV-*env *PCR was highly sensitive when tested on several blood samples obtained from HTLV infected individuals as well as from STLV infected monkeys. As a highly conserved control sequence we also amplified and sequenced intrahost fragments corresponding to a 219 bp fragment of the HTLV-1 *tax *gene. For our study, we used degenerate PCR primers in order to match all different sequences present in the template. One μg of DNA from fresh PBMCs for each sample was amplified by nested PCR using the primers and cycling conditions as follows(using standard abbreviations for degenerate positions) : 83VS, 5' TAYBTATTYCCNCATTGG 3'; and 240VAS, 5' RTANAGNACRTGCCA 3', located in the Y/LFPHW motif and WHVLY motif, respectively (positions 5452 to 5926 in the ATK-1 reference strain [34]) for the first amplification round and 83VS and 146VAS, 5' NACYTCYTGRGTRAARTT 3', the latter corresponding to the NFTQEV motif, for the second round of amplification. Touch-down PCR was performed using High Fidelity Platinium^® ^*Taq *DNA Polymerase (Invitrogen) including a hot start (94°C for 2 min), with the following cycle conditions: 26 cycles of 94°C for 30 s, 50°C decreasing by 0.5°C per cycle, and 72°C for 45 s; this was followed by 12 cycles of 94°C for 30 s, 48°C for 30 s, and 72°C for 45 s, with a final elongation at 72°C for 5 min before cooling to 4°C. Then, 1/15th of the first PCR volume was used as the source of templates in a semi-nested amplification performed with the same cycling conditionsexcept for the annealing step (50°C decreasing 0.5°C per cycle for the first 10 cycles followed by 30 cycles at 50°C). The *tax-rex *fragments were amplified using previously described generic primers and PCR conditions [35]. PCR amplification products were then purified by gel extraction and cloned into pGEM-TEasy vector (Promega). Recombinant plasmids were sequenced using cycle sequencing and dye terminator methodologies (DYEnamic ET Terminator Cycle Sequencing Kit [Amersham Biosciences]) on an automated sequencer (ABI Prism 310, Applied Biosystems). Fifty independent clones and 68 to 86 independent clones were sequenced for *tax *and *env *regions, respectively. Nucleotide and amino acid sequences (corresponding to the PCR fragment without primers) were aligned with ClustalW(1.7) [36], and analysis of the selective pressure was performed for all *env *sequences as described by Nei and Gojobori [37].

We used highly cross-reactive *tax *primer pairs, previously shown to match sequences from a variety of divergent HTLV and STLV strains, to amplify our samples. Sequencing and subsequent analyses of the corresponding 183 bp fragments of 50 independent PTLV-1 *tax *clones derived independently from one healthy carrier, one ATL patient and 2 macaques (Mac1 and Mac2), resulted in only a single nucleotide change in 2 clones. One change was observed in a clone derived from Mac1, while the second change was observed in a clone derived from the ATL patient sample. Each change translated into a different amino acid substitution when compared to the consensus sequence. Thus, in our experimental conditions, the frequency of bases changes that could be attributed to the *Taq *polymerase remained under 0.4%.

### Intrahost variations in PTLV-1 RBD sequences

**Figure 1:**
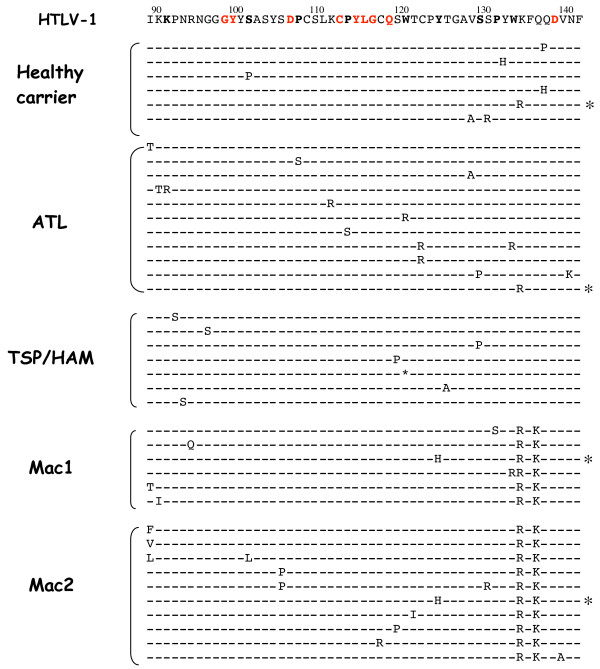
**Amino acid alignment of the RBD region in the SUgp46 of different variants isolated from 3 HTLV-1 infected patients and 2 STLV-1 naturally infected Celebes macaques**. Sequences are aligned with the dominant viral genotype found in the 3 HTLV-1 infected individuals. Dots indicate no change in amino acid and the asterisk denotes a stop codon. Numbers at the top represent the position in Env in reference to the ATK-1 sequence [34]. Amino acids in bold refer to conserved positions found on the multiple alignments of available PTLV types. Amino acids in red refer to positions that remain conserved among our variants. Identical variants found in different hosts are indicated by an asterisk (*) on the right side of the sequence.

In contrast to the apparently homogeneous viral population observed after *tax *sequencing, a significant degree of variability was seen with *env*. Indeed, there was an important sequence heterogeneity within each isolate, indicative of a quasispecies nature of HTLV-1/STLV-1 infections, as revealed by intrahost variations in the Env RBD (Figure 1). Overall, the different point mutations appeared randomly distributed throughout the fragment. The maximum pairwise distances within each group of quasispecies were 1.8%, 2.5% and 3.8% in the TSP/HAM, ATL and asymptomatic HTLV-1 infected patients, respectively, and ranged from 1.8% to 4.4% in STLV-1 macaques, reflecting an equal range of quasispecies diversity in the two host species (Table 1). In each sample, six to 11 intrahost variants were identified at the nucleotide level. For example, among the 77 clones sequenced from the TSP/HAM patient, 16 clones had one or more point mutations in the RBD, amongst which 37.5% led to an amino acid change. Two variants in the TSP/HAM patient presented a G-to-A mutation that translates into a stop codon at position 120 of the gp46 (Figure 1). Frequencies of nucleotide substitutions in the three HTLV-1 patients were comparable to those of the 2 STLV-1 macaques. Overall, we recorded 88 point mutations, with T-to-C transitions more frequently observed than A-to-G, as previously reported by others for *env *variants [38]. Altogether, these 88 point mutations led to 46 amino acid substitutions (Table 1). With the low percentage of background *Taq *polymerase error estimated with *tax*, it was clear that the majority of the RBD variants observed in our study occurred *in vivo *in both the simian and human natural hosts.

**Table 1:** Analyses of the variations observed in the RBD-encoding region of the *env *gene in 3 HTLV-1 infected individuals and 2 STLV-1 naturally infected Celebes macaques.

***Samples***	***Number of clones***	***Number of substitutions***	***Substitutions/clone***	***Nonsynonymous substitutions/substitutions***	***Number of variants***	***dn/ds***
Healthy carrier	71	17	0.24	0.41	6	0.298
ATL	86	21	0.24	0.66	11	0.292
TSP/HAM	77	17	0.22	0.35	6*	0.323
Mac1	68	10	0.15	0.7	7	0.295
Mac2	78	23	0.29	0.54	10	0.303

* Substitutions leading to a stop codon

The rate of nonsynonymous changes per nonsynonymous site (*dn*) and the rate of synonymous changes per synonymous site (*ds*) were calculated for the RBD sequences of each patient and macaque. A nonsynonymous substitution rate higher than the synonymous substitution rate indicates positive selection for advantageous mutations, whereas a nonsynonymous rate lower than the synonymous rate indicates « purifying selection » that prevents the spread of detrimental mutations [39,40]. A significantly higher rate of nonsynonymous substitutions was observed with the ATL sample as compared to the TSP/HAM sample. Although this might be related to the infection history and clinical features of the two patients, a larger series of samples will be required to assess this initial observation. The *dn/ds *ratio we calculated were relatively low (< 0.5) for all blood samples, irrespective of the host species and the clinical status. Therefore, despite the significant level of intrahost variations observed in the RBD sequences, strong constraints against sequence variation prevailed in the RBD region of the PTLV-1 *env *genes.

### Conserved residues in RBD

Our multiple alignments of the amplified region of the SU RBD showed that several residues such as, K91, S101, D106, Q118, W120, Y124, S129, P131, W133 and D138 or motifs such as, G98Y99 and C112PYLG116 are highly conserved between all HTLV and STLV virus strains available from Genbank. Comparative analyses of all our RBD variants highlighted a fewer number of positions with conserved residues, including D106, D138, GY and CPYLG (Figure 1). Y114 in the CPYLG motif and D106, previously described as important for HTLV Env receptor binding were conserved in all our variants. A P113 to S change observed within the highly conserved CPYLG motif might have either derived from *Taq *errors or represented a *bona fide *mutant. It would, therefore, be interesting to test whether this mutation affected different Env functions.

In summary, our results illustrate the diversity of proviral sequences that coexist within the *env *RBD. These *in vivo *findings suggest that there is an ongoing viral replication in PTLV-1 infected hosts, regardless of the clinical status and the host species. In light of these results that unveil significant intrahost variations in the *env *RBD region and not in the *tax *region, it will be of interest to evaluate intrahost variability, ideally at different stages of infection, within other regions of the viral genome.

Some of the variants identified here have never been described previously. Moreover, several variants were identified in unrelated samples (variants common to the asymptomatic and ATL donors, and variants common to the two macaques), suggestive of a robust selectivity conferred *in vivo *by these mutations (see legend to Figure 1).

Interestingly, the low degree of *env *sequence variation found between isolates does not reflect the significant degree of *env *sequence variation found within individuals. However, the same dominant HTLV-1 sequence was found independently in the three unrelated infected patients and the two STLV-1 macaques, in agreement with a strong positive pressure on this highly conserved consensus. Altogether, our results point to the selective transmission of an optimally adapted form, rather than to an absence of replication or to a stricter polymerase fidelity of PTLV-1.

Importantly, our new highly sensitive *env *PCR protocol, based on degenerate primers matching conserved motifs in the RBD, allows the detection of all known PTLV types (data not shown). This property will help elucidate further the detection of undescribed PTLV divergent variants as well as that of potentially undescribed PTLV types which would be masked under conventional PTLV PCR screening.

## Abbreviations used

Env: envelope glycoprotein

HTLV : Human T-cell lymphotropic virus

STLV : Simian T-cell lymphotropic virus

PTLV:Primate T-cell lymphotropic virus

MLV: Murine leukemia virus

nt: nucleotide

LTR: Long Terminal Repeat

PCR: Polymerase Chain Reaction

RBD: receptor-binding domain

SU: Env extracellular surface component

## Nucleotide accession number

The *env *accession number for the sequences determined in this study are: Genbank DQ530557 to DQ530596.

## Competing interests

The author(s) declare that they have no competing interests.

## Authors' contributions

FJK set up the initial design and experiments and participated to the writing of the manuscript. ML performed some of the PCR, cloning and sequencing experiments. AG and SG provided some of the DNA samples and corrected the final draft of the manuscript. JLB participated to the design of the study, helped with the interpretation of the data and corrected the manuscript. MS initiated the project, co-participated in the design of the study, co-coordinated its realization and co-wrote the manuscript. VC was the principal designer and experimentator of this study, coordinated its realization, wrote the first draft of the manuscript and co-wrote the following versions. All authors read and approved the final manuscript.
